# COVID-19 Infection Negative in Nasopharyngeal Swabs but Suspected in Computed Tomography and Confirmed in Bronchoalveolar Lavage Material

**DOI:** 10.1155/2021/6627207

**Published:** 2021-04-20

**Authors:** Robert Chrzan, Tadeusz Popiela, Maciej Małecki, Jan Skupień, Amira Bryll, Anna Grochowska

**Affiliations:** ^1^Jagiellonian University Medical College, Chair of Radiology, Kraków, Poland; ^2^Jagiellonian University Medical College, Department of Metabolic Diseases, Kraków, Poland

## Abstract

We present a case of a patient with clinical symptoms of pneumonia, negative in several polymerase chain reaction COVID-19 tests from nasopharyngeal swabs but suspected in computed tomography and finally confirmed in bronchoalveolar lavage material.

## 1. Introduction

High hopes were placed for using diagnostic imaging in the detection of positive cases in the early stages of the COVID-19 pandemic, but it was proved that only polymerase chain reaction (PCR) laboratory test of nasopharyngeal swab material should be the basic method of verifying COVID-19. However, in clinical practice, it turns out that this method also has limitations.

## 2. Case Presentation

In April 2020, a 59-year-old man was admitted to the university hospital for left-sided pneumonia treatment from home quarantine due to his wife's COVID-19 diagnosis.

According to the patient's history, he had a fever up to 40°C, mainly at night, pain when swallowing, and a dry cough. Nasopharyngeal swabs for the COVID-19 PCR tests, performed several times in a regional hospital and in the university hospital, were negative. In the laboratory tests on the day of admission, lymphopenia, moderately elevated C-reactive protein (CRP), and procalcitonin were found.

On chest X-ray, pneumonia in the left lung was suspected. Therefore, amoxicillin with clavulanic acid (1 g, twice a day) was included in the treatment.

Despite the antibiotic therapy, feverish conditions up to 39°C persisted, and increasing inflammatory parameters were observed.

**Figure 1:**
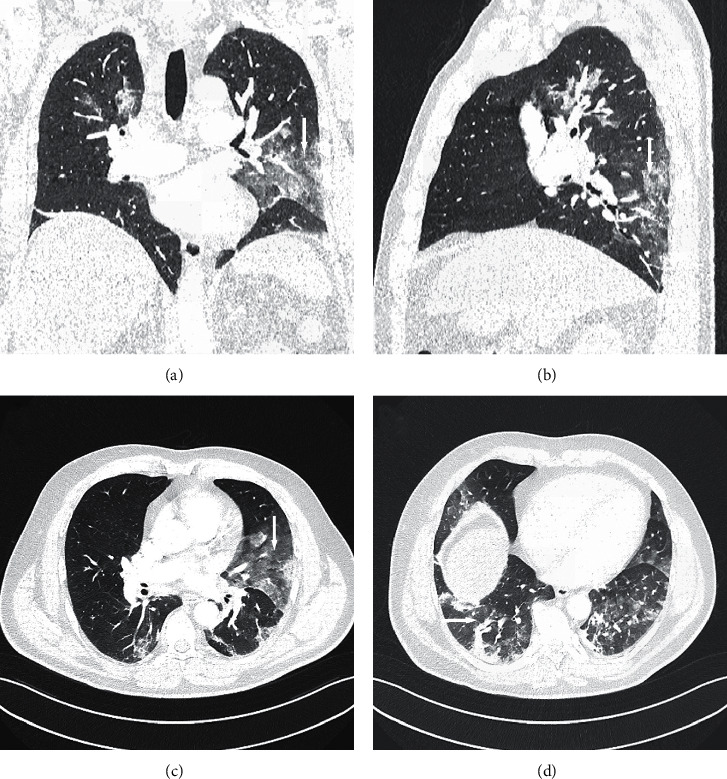
Chest computed tomography: (a) reconstruction in the coronal plane—“ground glass” opacities in both lungs; (b) reconstruction in the sagittal plane—“ground glass” opacities in the lower left lobe; (c) axial plane—“ground glass” opacities with a predominance of the left side; (d) axial plane—“ground glass” opacities and small areas of consolidation.

Chest angio-computed tomography (CT) was performed because of pulmonary embolism suspicion, but it was not confirmed. However, interstitial “ground glass” opacities were found in both the lungs (Figure 1(a)), especially in the middle and lower parts (Figure 1(b)), with left-side predominance (Figure 1(c)). Moreover, “crazy paving” zones, small areas of consolidation (Figure 1(d)) with fibrosis and small bronchiectasis in basal segments of the lower lobes, and reactive mediastinal lymph nodes were also found.

After pulmonary consultation, a bronchoscopy was performed with bronchoalveolar lavage (BAL) and collection of material was done for COVID-19 testing, microbiological cultures, and BACTEC. Eventually, in the material from BAL, COVID-19 was confirmed by PCR. No superimposed bacterial or fungal infection was found on the BAL studies.

The above pattern of “ground glass” typically occurs in COVID-19 pneumonia [1, 2], as well as other viral (influenza, H1N1, SARS, and MERS) and “atypical” (*Mycoplasma pneumoniae*, *Chlamydia pneumoniae*, *Legionella pneumophila*, and *Coxiella burnetii*) pneumonia.

The patient was then treated with ceftriaxone (1 g i.v., twice a day), low-molecular-weight heparin, and passive oxygen therapy. No steroids, convalescent plasma, or remdesivir therapy was used. Resolution of respiratory failure, regression of radiological changes, and normalization of inflammatory markers and D-dimer were obtained. Control swabs were negative for COVID-19. The patient refused a control bronchoscopy with BAL and COVID-19 testing.

The patient was discharged home in good condition.

## 3. Discussion

According to the current recommendations of radiological societies (American College of Radiology [3] and British Thoracic Imaging Society [4]), CT should not be used as a screening test or as the first-line test in the diagnosis of COVID-19 infection because of the limited specificity, as these symptoms may occur in infections of other etiologies.

In a meta-analysis performed by Khatami et al. [5], based on 60 studies and 5744 patients, the overall sensitivity, specificity, positive predictive value, and negative predictive value of chest CT scan in the detection of COVID-19 infection were 87%, 46%, 69%, and 89%, respectively.

Another meta-analysis by Kim et al. [6], based on 63 studies and 6218 patients, found a pooled sensitivity of 94% and specificity of 37% for chest CT scan in the detection of COVID-19 infection.

The PCR laboratory test of nasopharyngeal swab remains the basic method of verifying COVID-19. However, a clinical practice shows that there is a group of patients with a significant risk of COVID-19 infection (contact with infected persons and several clinical features of infection), in whom the initial nasopharyngeal swab does not confirm COVID-19 infection, but it is positively verified in subsequent PCR assessments. Several authors confirmed that the sensitivity of PCR might not be optimal at the beginning of the disease [7–9].

It may be useful to perform chest HRCT (high-resolution computed tomography) in such patients, while the analysis of such scans can be supported by dedicated computer programs, using deep machine learning techniques (“artificial intelligence”) [10].

In case of negative initial nasopharyngeal swab, but COVID-19 infection suspicion in chest CT, it is suggested to repeat the swab. However, other authors also confirm the possibility of several negative nasopharyngeal swabs, but the final confirmation of COVID-19 infection in PCR from BAL material [11, 12]. Mencarini et al. [12] reported a rate of 37.2% in the detection of COVID-19 on BAL in patients with suspected infection.

## 4. Conclusions

1. CT should not be typically used as a screening test or as the first-line test in the diagnosis of COVID-19 infection because of the limited specificity
2. The PCR laboratory test of nasopharyngeal swab material remains the basic method of verifying COVID-19
3. However, in a few patients, the initial nasopharyngeal swab does not confirm COVID-19 infection, but the symptoms may be visible in CT and it may be positively verified in one of the following swabs or only in the PCR test of BAL material

## Data Availability

The laboratory and radiological variables and outcome data will be made available upon request.

## References

[1] Pan F., Ye T., Sun P. (2020). Time course of lung changes at chest CT during recovery from coronavirus disease 2019 (COVID-19). *Radiology*.

[2] Rogalska-Płońska M., Kuźmicz A., Łapiński T. W., Flisiak R. (2020). Computed tomography changes in lungs of COVID-19 patients. *Polish Archives of Internal Medicine*.

[3] American College of Radiology (2020). ACR recommendations for the use of chest radiography and computed tomography (CT) for suspected COVID-19 infection. https://www.acr.org/Advocacy-and-Economics/ACR-Position-Statements/Recommendations-for-Chest-Radiography-and-CT-for-Suspected-COVID19-Infection.

[4] Rodrigues J. C. L., Hare S. S., Edey A. (2020). An update on COVID-19 for the radiologist-a British society of thoracic imaging statement. *Clinical Radiology*.

[5] Khatami F., Saatchi M., Zadeh S. S. T. (2020). A meta-analysis of accuracy and sensitivity of chest CT and RT-PCR in COVID-19 diagnosis. *Scientific Reports*.

[6] Kim H., Hong H., Yoon S. H. (2020). Diagnostic performance of CT and reverse transcriptase polymerase chain reaction for coronavirus disease 2019: a meta-analysis. *Radiology*.

[7] Long C., Xu H., Shen Q. (2020). Diagnosis of the Coronavirus disease (COVID-19): rRT-PCR or CT?. *European Journal of Radiology*.

[8] Xie X., Zhong Z., Zhao W., Zheng C., Wang F., Liu J. (2020). Chest CT for typical coronavirus disease 2019 (COVID-19) pneumonia: relationship to negative RT-PCR testing. *Radiology*.

[9] Fang Y., Zhang H., Xie J. (2020). Sensitivity of chest CT for COVID-19: comparison to RT-PCR. *Radiology*.

[10] Li L., Qin L., Xu Z. (2020). Using artificial intelligence to detect COVID-19 and community-acquired pneumonia based on pulmonary CT: evaluation of the diagnostic accuracy. *Radiology*.

[11] Gualano G., Musso M., Mosti S. (2020). Usefulness of bronchoalveolar lavage in the management of patients presenting with lung infiltrates and suspect COVID-19-associated pneumonia: a case report. *International Journal of Infectious Diseases*.

[12] Patrucco F., Albera C., Bellocchia M. (2020). SARS-CoV-2 Detection on Bronchoalveolar Lavage: An Italian Multicenter experience. *Respiration*.

